# Myeloid Sarcoma of the Parotid Gland and Stomach Presenting with Obstructive Jaundice: A Rare Presentation

**DOI:** 10.4274/tjh.galenos.2019.2018.0302

**Published:** 2019-08-02

**Authors:** Sugeeth M. Thambi, Sreejith G. Nair, Rony Benson, Jayasudha A. Vasudevan, Rekha A. Nair

**Affiliations:** 1 Regional Cancer Centre, Department of Medical Oncology, Thiruvananthapuram, India; 2 Regional Cancer Centre, Department of Pathology, Thiruvananthapuram, India

**Keywords:** Myeloid sarcoma, Parotid gland, Stomach

## To the Editor,

Myeloid sarcoma (MS) is the extramedullary deposit of immature myeloid cells and disrupts the normal tissue architecture [1]. MS commonly occurs in the skin, central nervous system, eyes, and testes. Gastrointestinal involvement is common [2,3]. Here we present a case of isolated MS of the parotid and stomach presenting with jaundice.

**Figure 1:**
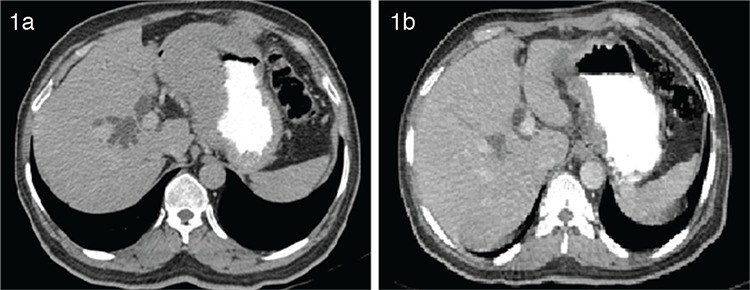
Contrast-enhanced computed tomography of the neck, chest, and abdomen showing intrahepatic biliary radicle dilatation and stomach wall thickening involving the cardia and lesser curvature of the stomach (a) and post-induction scan showing significant reduction in the stomach wall thickening and resolution of intrahepatic biliary radicle dilatation (b).

A 55-year-old male was evaluated with swelling of the right parotid gland for two months. Fine-needle aspiration was suggestive of a parotid neoplasm and the patient underwent a right-sided total parotidectomy. Post-op histopathological examination was suggestive of non-Hodgkin’s lymphoma. While the patient was recovering, he developed jaundice. Liver function tests showed bilirubin of 5.3 mg/dL (direct: 4.2 mg/dL). Contrast-enhanced computed tomography of the neck, chest, and abdomen was performed, which showed irregular soft tissue thickening in the parotid bed along with an enlarged enhancing left level IB nodal area (21x12 mm). The abdomen showed intrahepatic biliary radicle dilatation with a soft tissue nodule at the porta. There was also soft tissue thickening involving the cardia and lesser curvature of the stomach along with multiple enlarged perigastric nodes (Figure 1).

**Figure 2:**
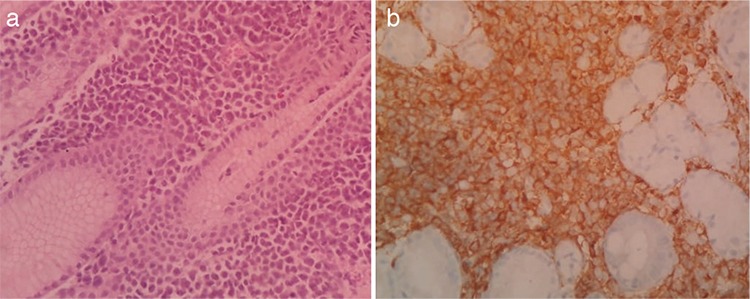
(a) Hematoxylin and eosin results showing medium to large atypical cells with scanty cytoplasm and irregular nuclear membranes; (b) tumor cells positive for myeloperoxidase.

Peripheral smear and bone marrow studies were normal. Review of the parotidectomy specimen showed a neoplasm composed of atypical medium to large cells. Tumor cells were myeloperoxidase-positive, CD33-positive, CD43 focal-positive, and CD68-negative and were compatible with MS (Figure 2). During work-up bilirubin increased to 20 mg/dL and the patient underwent percutaneous transhepatic biliary drainage. Upper gastrointestinal endoscopy was suggestive of mucosal irregularity involving the cardia and lesser curvature of the stomach. Endoscopic guided biopsy from the lesion was suggestive of MS. The patient’s bilirubin normalized after stenting.

The patient was scheduled for 7+3 induction (7 days of cytarabine at 100 mg/m^2^ as a 24-hour infusion along with 3 days of daunorubicin at 60 mg/m^2^). Post-induction reevaluation was done and contrast-enhanced computed tomography showed no significant lymph nodes, with significant reduction in the gastric and duodenal wall thickening along with resolution of the intrahepatic biliary radicle dilatation. The patient was scheduled for consolidation with high-dose cytarabine and received 3 cycles. He remained on follow-up after the completion of 3 cycles.

Isolated MS usually does not produce any specific symptoms besides the local symptoms of the organ involved. Local imaging is usually warranted in the form of computed tomography or magnetic resonance imaging [4]. Bone marrow study is also warranted to confirm isolated MS as most cases occur in patients with AML. Systemic therapy is warranted in such cases where patients receive induction chemotherapy similar to AML, as in our case [5]. The 5-year survival in patients with MS is about 20% and the use of chemotherapy has been associated with better survival [6]. There are reports that malignant cells in chloroma may evade immune surveillance and thus have a higher chance of survival. Another contributing factor to immune escape is the partial loss of several human leukocyte antigen class I genes [7].
